# Supplementary material for: Effectiveness of Colorectal Cancer (CRC) Screening on All-Cause and CRC-Specific Mortality Reduction: A Systematic Review and Meta-Analysis
Source: Cancers (Basel). 2023 Mar 24;15(7):1948. doi: 10.3390/cancers15071948 (PMC10093633; doi:10.3390/cancers15071948)
Supplement: Supplementary file 1 [file cancers-15-01948-s001.zip › cancers-2249950-supplementary.pdf]

Supplementary Materials

Table S1. Literature search strategy.

**Search number**	**Query on PubMed**
**RCT**	
#6	(#1) AND (#2) AND (#3) AND (#4) Filters: from 2006/1/1 - 2022/07/31
#5	(#1) AND (#2) AND (#3) AND (#4)
#4	("Randomized Controlled Trial" [Publication Type] OR "Randomized Controlled Trials as Topic"[Mesh] OR random*[tiab] OR trial*[ti])
#3	("Mortality"[Mesh] OR "mortality" [Subheading] OR "Cost-Benefit Analysis"[Mesh] OR effectiveness[tiab] OR life-year*[tiab] OR year-of-life[tiab] OR mortalit*[tiab] OR death*[tiab] OR surviv*[tiab] OR ((cost[tiab] OR costs[tiab]) AND (benefit*[tiab] OR effect*[tiab])) OR economic-anal*[tiab])
#2	("Early Detection of Cancer"[Mesh] OR "Mass Screening"[Mesh:NoExp] OR screen*[tiab] OR "early detect*"[tiab] OR "fecal immunochemical test*"[tiab] OR "faecal immunochemical test*"[tiab] OR "fecal occult blood test*"[tiab] OR "faecal occult blood test*"[tiab] OR colonoscop*[tiab] OR sigmoidoscop*[tiab])
#1	("Colorectal Neoplasms"[Mesh] OR "colorectal cancer*"[tiab] OR "colon cancer*"[tiab] OR "rectum cancer*"[tiab] OR "rectal cancer*"[tiab] OR "colonic cancer*"[tiab] OR "colorectal tumo*"[tiab] OR "colon tumo*"[tiab] OR "rectum tumo*"[tiab] OR "rectal tumo*"[tiab] OR "colonic tumo*"[tiab] OR "colorectal neoplas*"[tiab] OR "colon neoplas*"[tiab] OR "rectum neoplas*"[tiab] OR "rectal neoplas*"[tiab] OR "colonic neoplas*"[tiab] OR "bowel cancer*"[tiab])
**Modelling study**	
#6	(#1) AND (#2) AND (#3) AND (#4) Filters: from 2016/1/1 - 2022/07/31
#5	(#1) AND (#2) AND (#3) AND (#4)
#4	("Models, Theoretical"[Mesh] OR "Computer Simulation"[Mesh] OR "Markov Chains"[Mesh] OR model[tiab] OR models[tiab] OR modelling[tiab] OR modeling[tiab] OR simulat*[tiab] OR microsimulat*[tiab] OR markov[tiab])
#3	("Mortality"[Mesh] OR "mortality" [Subheading] OR "Cost-Benefit Analysis"[Mesh] OR effectiveness[tiab] OR life-year*[tiab] OR year-of-life[tiab] OR mortalit*[tiab] OR death*[tiab] OR surviv*[tiab] OR ((cost[tiab] OR costs[tiab]) AND (benefit*[tiab] OR effect*[tiab])) OR economic-anal*[tiab])
#2	("Early Detection of Cancer"[Mesh] OR "Mass Screening"[Mesh:NoExp] OR screen*[tiab] OR "early detect*"[tiab] OR "fecal immunochemical test*"[tiab] OR "faecal immunochemical test*"[tiab] OR "fecal occult blood test*"[tiab] OR "faecal occult blood test*"[tiab] OR colonoscop*[tiab] OR sigmoidoscop*[tiab])
#1	("Colorectal Neoplasms"[Mesh] OR "colorectal cancer*"[tiab] OR "colon cancer*"[tiab] OR "rectum cancer*"[tiab] OR "rectal cancer*"[tiab] OR "colonic cancer*"[tiab] OR "colorectal tumo*"[tiab] OR "colon tumo*"[tiab] OR "rectum tumo*"[tiab] OR "rectal tumo*"[tiab] OR "colonic tumo*"[tiab] OR "colorectal neoplas*"[tiab] OR "colon neoplas*"[tiab] OR "rectum neoplas*"[tiab] OR "rectal neoplas*"[tiab] OR "colonic neoplas*"[tiab] OR "bowel cancer*"[tiab])
**Search number**	**Query on Embase**
**RCT**	
#7	(#1) AND (#2) AND (#3) AND (#4) NOT (#5) Filters: from 2006/1/1 - 2022/07/31
#6	(#1) AND (#2) AND (#3) AND (#4) NOT (#5)
#5	'conference abstract'/it
#4	('randomized controlled trial'/exp OR random*:ab,ti,kw OR trial*:ti)
#3	('mortality'/exp OR 'survival'/exp OR 'cost benefit analysis'/exp OR 'cost effectiveness analysis'/exp OR (effectiveness OR "life-year*" OR "year-of-life" OR mortalit* OR death* OR surviv* OR ((cost OR costs) AND (benefit* OR effect*)) OR "economic-anal*"):ab,ti,kw)

#2	('early cancer diagnosis'/exp OR 'mass screening'/de OR 'cancer screening'/exp OR 'screening test'/exp OR 'occult blood test'/exp OR 'colonoscopy'/exp OR 'sigmoidoscopy'/exp OR (screen* OR "early detect*" OR "fecal immunochemical test*" OR "faecal immunochemical test*" OR "fecal occult blood test*" OR "faecal occult blood test*" OR colonoscop* OR sigmoidoscop*):ab,ti,kw)
#1	('colorectal tumor'/exp OR 'colon tumor'/exp OR 'rectum tumor'/exp OR ("colorectal cancer*" OR "colon cancer*" OR "rectum cancer*" OR "rectal cancer*" OR "colonic cancer*" OR "colorectal tumo*" OR "colon tumo*" OR "rectum tumo*" OR "rectal tumo*" OR "colonic tumo*" OR "colorectal neoplas*" OR "colon neoplas*" OR "rectum neoplas*" OR "rectal neoplas*" OR "colonic neoplas*" OR "bowel cancer*"):ab,ti,kw)
**Modelling study**	
#7	(#1) AND (#2) AND (#3) AND (#4) NOT (#5) Filters: from 2016/1/1 - 2022/07/31
#6	(#1) AND (#2) AND (#3) AND (#4) NOT (#5)
#5	'conference abstract'/it
#4	('model'/de OR 'theoretical model'/exp OR 'simulation'/exp OR 'Markov chain'/exp OR (model OR models OR modeling OR modelling OR simulat* OR microsimulat* OR markov):ab,ti,kw)
#3	('mortality'/exp OR 'survival'/exp OR 'cost benefit analysis'/exp OR 'cost effectiveness analysis'/exp OR (effectiveness OR "life-year*" OR "year-of-life" OR mortalit* OR death* OR surviv* OR ((cost OR costs) AND (benefit* OR effect*)) OR "economic-anal*"):ab,ti,kw)
#2	('early cancer diagnosis'/exp OR 'mass screening'/de OR 'cancer screening'/exp OR 'screening test'/exp OR 'occult blood test'/exp OR 'colonoscopy'/exp OR 'sigmoidoscopy'/exp OR (screen* OR "early detect*" OR "fecal immunochemical test*" OR "faecal immunochemical test*" OR "fecal occult blood test*" OR "faecal occult blood test*" OR colonoscop* OR sigmoidoscop*):ab,ti,kw)
#1	('colorectal tumor'/exp OR 'colon tumor'/exp OR 'rectum tumor'/exp OR ("colorectal cancer*" OR "colon cancer*" OR "rectum cancer*" OR "rectal cancer*" OR "colonic cancer*" OR "colorectal tumo*" OR "colon tumo*" OR "rectum tumo*" OR "rectal tumo*" OR "colonic tumo*" OR "colorectal neoplas*" OR "colon neoplas*" OR "rectum neoplas*" OR "rectal neoplas*" OR "colonic neoplas*" OR "bowel cancer*"):ab,ti,kw)
**Search number**	**Query on Web of Science**
**RCT**	
#6	(#1) AND (#2) AND (#3) AND (#4) Filters: from 2006/1/1 - 2022/07/31
#5	(#1) AND (#2) AND (#3) AND (#4)
#4	TS=("Randomized Controlled*" OR random* OR trial*)
#3	TS=(effectiveness OR life-year* OR year-of-life OR mortalit* OR death* OR surviv* OR ((cost OR costs) AND (benefit* OR effect*)) OR economic-anal*)
#2	TS=(screen* OR "early detect*" OR "fecal immunochemical test*" OR "faecal immunochemical test*" OR "fecal occult blood test*" OR "faecal occult blood test*" OR colonoscop* OR sigmoidoscop*)
#1	TS=("colorectal cancer*" OR "colon cancer*" OR "rectum cancer*" OR "rectal cancer*" OR "colonic cancer*" OR "colorectal tumo*" OR "colon tumo*" OR "rectum tumo*" OR "rectal tumo*" OR "colonic tumo*" OR "colorectal neoplas*" OR "colon neoplas*" OR "rectum neoplas*" OR "rectal neoplas*" OR "colonic neoplas*" OR "bowel cancer*")
**Modelling study**	
#6	(#1) AND (#2) AND (#3) AND (#4) Filters: from 2016/1/1 - 2022/07/31
#5	(#1) AND (#2) AND (#3) AND (#4)
#4	TS=(model OR models OR modeling OR modelling OR simulat* OR microsimulat* OR markov)
#3	TS=(effectiveness OR life-year* OR year-of-life OR mortalit* OR death* OR surviv* OR ((cost OR costs) AND (benefit* OR effect*)) OR economic-anal*)
#2	TS=(screen* OR "early detect*" OR "fecal immunochemical test*" OR "faecal immunochemical test*" OR "fecal occult blood test*" OR "faecal occult blood test*" OR colonoscop* OR sigmoidoscop*)

#1	TS=("colorectal cancer*" OR "colon cancer*" OR "rectum cancer*" OR "rectal cancer*" OR "colonic cancer*" OR "colorectal tumo*" OR "colon tumo*" OR "rectum tumo*" OR "rectal tumo*" OR "colonic tumo*" OR "colorectal neoplas*" OR "colon neoplas*" OR "rectum neoplas*" OR "rectal neoplas*" OR "colonic neoplas*" OR "bowel cancer*")
**Search number**	**Query on Cochrane Library**
#2	Filters: from 2006/1/1 - 2022/07/31
#1	("colorectal cancer*" OR "colon cancer*" OR "rectum cancer*" OR "rectal cancer*" OR "colonic cancer*" OR "colorectal tumo*" OR "colon tumo*" OR "rectum tumo*" OR "rectal tumo*" OR "colonic tumo*" OR "colorectal neoplas*" OR "colon neoplas*" OR "rectum neoplas*" OR "rectal neoplas*" OR "colonic neoplas*" OR "bowel cancer*") AND (screen* OR "early detect*" OR "fecal immunochemical test*" OR "faecal immunochemical test*" OR "fecal occult blood test*" OR "faecal occult blood test*" OR colonoscop* OR sigmoidoscop*) AND (effectiveness OR life-year* OR year-of-life OR mortalit* OR death* OR surviv* OR ((cost OR costs) AND (benefit* OR effect*)) OR economic-anal*)

Box S1. Selection criteria for articles.

Inclusion criteria:

1. Population: General population at average risk for colorectal cancer (CRC).
2. Study type: Simulation modelling study (model-based cost-effectiveness study) or randomized controlled trials comparing CRC screening with no screening.
3. Outcome: Provide outcome of interest on the survival, death number, and mortality reduction of CRC screening.
4. Original studies (peer-reviewed paper or report).
5. Published in English.

Exclusion criteria:

1. Not related to CRC screening.
2. No comparison between CRC screening and no screening.
3. Not a multi-use simulation modelling study (model-based cost-effectiveness study) or a randomized controlled trial.
4. Not a general population (e.g., CRC patients, CRC middle/high-risk population)
5. CRC specific mortality reduction, all-cause mortality reduction, number of deaths prevented, or mortality rate not reported. (LGY and QALY were considered in the title and abstract screening).
6. Not providing detailed numbers in main text or supplementary files for outcomes we are interested in, only showing in figure.
7. Not including any screening test commonly used, or not using any common screening strategy. (Annual FIT/FOBT, Biennial FIT/FOBT, Triennial FIT/FOBT, 10-yearly colonoscopy, 5-yearly sigmoidoscopy, Single sigmoidoscopy/colonoscopy)
8. Not original research.
9. It’s a meta-analysis, systematic review, or review.
10. It’s a research protocol or study design.
11. It’s a screening program’s implementation analysis and report and did not provide original data.
12. Studies published in conference abstract or editorial.
13. Studies published in languages other than English.

Box S2. Qualitative assessment framework on model characteristics.

- The modelling approach was evaluated from the statements of the adenoma/tumor progression, adenoma pathway, main sources of input data and assumptions, calibration, and the sensitivity analyses for uncertain variables [47,94].
- Model parameters contained: simulated population all-cause mortality rate, adenoma and CRC incidence rate, adenoma/CRC transition probabilities, survival by cancer stage, dynamic or static cohort, sensitivity/specificity of CRC screening methods, and adherence rate.
- The definition of transparency of data sources/assumptions was a systematic assessment and selection of data/assumption.
- The external validation of the models was extracted from the report of publications. External validation was defined as comparison of model outputs to real-world or independent data results to verify the generalization of the model [47,95].

Table S2. Subgroup analysis of RCTs (Intention-to-treat analyses[a]).

**Subgroups**	**Pooled estimate**	**No. of studies**	I^2**(%)**	P_{sub}
Adherence				
CRC-specific mortality reduction by FOBT		*5*		
≥70%	0.78 (0.68, 0.89)	2	34	**0.03**
＜70%	0.92 (0.87, 0.98)	3	0	
Screening start age				
All-cause mortality reduction by FS		5		
50	1.17 (0.97, 1.41)	2	70	0.08
55	0.99 (0.97, 1.00)	3	0	
Risk of Bias				
All-cause mortality reduction by FOBT				
Low RoB	1.00 (0.99, 1.01)	3	0	0.30
Some concerns or high RoB	1.01 (0.99, 1.03)	2	0	
All-cause mortality reduction by FS				
Low RoB	0.99 (0.96, 1.01)	2	6	0.19
Some concerns or high RoB	1.09 (0.94, 1.28)	3	93	
CRC-specific mortality reduction by FOBT				
Low RoB	0.86 (0.75, 0.98)	3	73	0.55
Some concerns or high RoB	0.92 (0.76, 1.13)	2	56	
CRC-specific mortality reduction by FS				
Low RoB	0.72 (0.64, 0.81)	2	0	0.86
Some concerns or high RoB	0.66 (0.28, 1.57)	3	51	

[a] Intention-to-treat analyses: including all individuals as randomized. Not be adjusted by compliance.

Table S3. Subgroup analysis of RCTs on CRC-specific mortality reduction by FOBT (Compliance-adjusted analysis).

**Subgroups**	**Pooled estimate**	**No. of studies**	I^2**(%)**	P_{sub}
Scemario				
CRC-specific mortality reduction by FOBT		3		
Annual	0.65 (0.52, 0.81)	1	—	**0.045**
Biennial	0.84 (0.74, 0.94)	3	0	

Table S4. Quality assessment of included models.

**Model**	**Modelling approach:**<br>(1) Adenoma pathway<br>(2) Adenoma/tumor progression<br>(3) Sensitivity analyses<br>(4) Calibration	**Model parameters:**<br>(1) Simulated population all-cause mortality rate<br>(2) Adenoma and CRC incidence rate<br>(3) Adenoma/CRC transition<br>(4) Survival by cancer stage<br>(5) Dynamic or static cohort<br>(6) Sensitivity/specificity of screening methods<br>(7) Adherence rate	**Transparency of data sources/assumptions** (Yes/No)	**External validation** (Yes/No)	**Overall risk of bias** (Low/High)
CRC-SPIN [30]	(1) Adenoma-carcinoma sequence.<br>(2) All adenomas may progress to CRC<br>(3) Yes. Sensitivity/specificity of tests.<br>(4) Yes.	(1) 2009 US life table.<br>(2) SEER (1975-1979)<br>(3) Growth curve for adenoma, adenoma size at transition for transition to preclinical CRC, and time to transition for transition to clinical CRC.<br>(4) Yes. SEER.<br>(5) Dynamic cohort.<br>(6) Yes.<br>(7) Yes. Full adherence.	No.	Yes. UKFSST.	Low
SimCRC [30]	(1) Adenoma-carcinoma sequence.<br>(2) Progressive adenomas may progress to CRC.<br>(3) Yes. Sensitivity/specificity of tests.<br>(4) Yes.	(1) 2009 US life table.<br>(2) SEER (1975-1979)<br>(3) Time in each size category for adenoma, logistic function for transition to preclinical CRC, and time to transition for transition to clinical CRC.<br>(4) Yes. SEER.<br>(5) Dynamic cohort<br>(6) Yes.<br>(7) Yes. Full adherence.	No.	Yes. UKFSST.	Low
MISCAN-Colon [30]	(1) Adenoma-carcinoma sequence.<br>(2) Progressive adenomas may progress to CRC.<br>(3) Yes. Sensitivity/specificity of tests.<br>(4) Yes.	(1) 2009 US life table.<br>(2) SEER (1990-1994)<br>(3) Time in each size category for adenoma, overall transition probability for transition to preclinical CRC, and time to transition for transition to clinical CRC.<br>(4) Yes. SEER.<br>(5) Dynamic cohort.<br>(6) Yes.	No.	Yes. UKFSST, NORCCAP, and SCORE.	Low

		(7) Yes. Full adherence.			
ASCCA [28]	(1) Adenoma-carcinoma pathway and serrated pathway.<br>(2) Advanced adenoma may progress to CRC, regression to smaller size allowed, SSA may progress into CRC.<br>(3) Yes. Detection of serrated pathway; adherence; sensitivity/specificity of tests.<br>(4) Yes.	(1) Dutch Central Bureau for Statistics.<br>(2) Dutch COlonoscopy or CT COlonography for Screening (COCOS) trial and the Dutch cancer registry.<br>(3) Annual transition rate for each size category of adenoma, and for from polyp to CRC.<br>(4) Yes. Dutch Cancer Registry.<br>(5) Dynamic cohort.<br>(6) Yes.<br>(7) Yes.	No.	Yes. Two Dutch screening trials.	Low
CMOST [41]	(1) Adenomatous precursors and non-adenomatous pathway.<br>(2) All adenomas may progress to CRC.<br>(3) Yes. Detection by colonoscopy; prevalence; adherence; compliance surveillance; FIT sensitivity/specificity; colonoscopy risk.<br>(4) Yes.	(1) 2008 US life table.<br>(2) SEER (2005-2009).<br>(3) Transition probabilities for adenoma transforms to CRC.<br>(4) Yes. SEER (1991-2000).<br>(5) Dynamic cohort.<br>(6) Yes.<br>(7) Yes. Full adherence and real-world adherence.	No.	No.	High
CRC-AIM [40]	(1) Adenoma-carcinoma pathway.<br>(2) All adenomas may progress to CRC.<br>(3) Yes. Sensitivity/specificity of tests; adherence.<br>(4) No.	(1) 1900-2010 US life table.<br>(2) No.<br>(3) Adenomas growth rates based on location: non-linear growth curve. The cumulative probability of an adenoma transitioning to preclinical CRC: function of adenoma size, age at adenoma initiation, sex, and location of the adenoma.<br>(4) Yes. SEER (2000-2003).<br>(5) Dynamic cohort.<br>(6) Yes.<br>(7) Yes. Full adherence and real-world adherence.	No.	Yes. CRC-SPIN, SimCRC, and MISCAN-Colon models.	High

Decision analytic Markov cohort model [70]	(1) Adenoma-carcinoma pathway.<br>(2) Large polyp may progress to CRC.<br>(3) Yes. Sensitivity of tests.<br>(4) Yes.	(1) US life table.<br>(2) SEER and autopsy data.<br>(3) Annual transition rate for each size category of adenoma, and for from polyp to CRC.<br>(4) Yes. SEER.<br>(5) Dynamic cohort<br>(6) Yes.<br>(7) Yes. Full adherence and real-world adherence.	No.	Yes. Minnesota Colon Cancer Control Study, UKFSST, PLCO Trial, and SCORE.	Low
Policy1-Bowel [33]	(1) Adenoma-carcinoma pathway and the serrated pathway.<br>(2) Advanced adenoma and SSA may progress to CRC, regression to smaller size allowed in small and diminutive adenoma.<br>(3) Yes. Detection rate of colonoscopy; non-fatal Complication; Sensitivity/specificity of tests.<br>(4) Yes.	(1) Australian Institute of Health and Welfare (AIHW). General record of incidence of mortality (GRIM) books 2013: all causes combined.<br>(2) 2001 Australian Standard Population data.<br>(3) Adenoma size progression and regression rate, probability of developing high-grade dysplasia by adenoma size and advanced adenoma progress into stage 1 non-symptomatic cancer.<br>(4) Yes. Western Australia data.<br>(5) Dynamic cohort<br>(6) Yes.<br>(7) Yes. Full adherence and real-world adherence.	No.	Yes. SCORE and UKFSST.	Low
Multistate Markov model [74]	(1) Adenoma-carcinoma process.<br>(2) Advanced adenoma may progress to CRC.<br>(3) Yes. Annual transition rates; colonoscopy compliance; screening starting age.<br>(4) No.	(1) German population life tables 2010/2012<br>(2) German screening colonoscopy program (2003-2012, first time screening).<br>(3) Nationwide screening colonoscopy registry.<br>(4) German population-based case-control study on CRC screening with long-term mortality follow-up.<br>(5) Static cohort.<br>(6) Yes.<br>(7) Yes. Full adherence.	No.	Yes. KolosSal study & registry-based estimates of CRC incidence in Germany.	High

CRC: Colorectal cancer; CRC-SPIN: CRC Simulated Population Model for Incidence and Natural History; SimCRC: Simulation Model of Colorectal Cancer; MISCAN-Colon: MIcrosimulation SCreening ANalysis-Colon; ASCCA: Adenoma and Serrated pathway to Colorectal CAncer; CMOST: Microsimulation-based colon modeling open-source tool; CRC-AIM: Colorectal Cancer and Adenoma Incidence and Mortality Microsimulation Model; UKFSST: United Kingdom Flexible Sigmoidoscopy Screening Trial; NORCCAP: Norwegian Colorectal Cancer Prevention Trial; SCORE: Screening for COlon REctum trial; PLCO Trial: Prostate, Lung, Colorectal and Ovarian Cancer Screening Trial.

Table S5. Characteristics of simulation models on biennial gFOBT screening from age of 45–80 years.

Model	Study No.	Study	Simulate population	Screening age	Simulation period/Follow-up time (years)	Adherence rate in screening group (%)	CRC mortality of no screening group	CRC mortality of screening group
SimCRC	1	Knudsen, A. B. (2016) [31]	US	45-75	Lifetime	100%	28.0/1000	6.0/1000
			US	50-75	Lifetime	100%	28.0/1000	7.0/1000
			US	55-75	Lifetime	100%	28.0/1000	9.0/1000
			US	45-80	Lifetime	100%	28.0/1000	5.0/1000
			US	50-80	Lifetime	100%	28.0/1000	6.0/1000
			US	55-80	Lifetime	100%	28.0/1000	8.0/1000
	2	Meester, R. G. S. (2018) [36]	US White Female	45-75	Lifetime	100%	25.7/1000	5.7/1000
			US Black Female	45-75	Lifetime	100%	30.0/1000	8.0/1000
			US White Male	45-75	Lifetime	100%	31.0/1000	6.0/1000
			US Black Male	45-75	Lifetime	100%	27.0/1000	6.0/1000
			US White Female	50-75	Lifetime	100%	25.7/1000	6.7/1000
			US Black Female	50-75	Lifetime	100%	30.0/1000	10.0/1000
			US White Male	50-75	Lifetime	100%	31.0/1000	8.0/1000
			US Black Male	50-75	Lifetime	100%	27.0/1000	8.0/1000
			US White Female	55-75	Lifetime	100%	25.7/1000	7.7/1000
			US Black Female	55-75	Lifetime	100%	30.0/1000	11.0/1000
			US White Male	55-75	Lifetime	100%	31.0/1000	9.0/1000
			US Black Male	55-75	Lifetime	100%	27.0/1000	10.0/1000
			US White Female	45-80	Lifetime	100%	25.7/1000	4.7/1000
			US Black Female	45-80	Lifetime	100%	30.0/1000	7.0/1000
			US White Male	45-80	Lifetime	100%	31.0/1000	5.0/1000
			US Black Male	45-80	Lifetime	100%	27.0/1000	6.0/1000
			US White Female	50-80	Lifetime	100%	25.7/1000	5.7/1000
			US Black Female	50-80	Lifetime	100%	30.0/1000	8.0/1000
			US White Male	50-80	Lifetime	100%	31.0/1000	6.0/1000

			US Black Male	50-80	Lifetime	100%	27.0/1000	7.0/1000
			US White Female	55-80	Lifetime	100%	25.7/1000	6.7/1000
			US Black Female	55-80	Lifetime	100%	30.0/1000	10.0/1000
			US White Male	55-80	Lifetime	100%	31.0/1000	8.0/1000
			US Black Male	55-80	Lifetime	100%	27.0/1000	9.0/1000
CRC-SPIN	1	Knudsen, A. B. (2016) [31]	US	45-75	Lifetime	100%	27.0/1000	7.0/1000
			US	50-75	Lifetime	100%	27.0/1000	8.0/1000
			US	55-75	Lifetime	100%	27.0/1000	9.0/1000
			US	45-80	Lifetime	100%	27.0/1000	6.0/1000
			US	50-80	Lifetime	100%	27.0/1000	6.0/1000
			US	55-80	Lifetime	100%	27.0/1000	8.0/1000
MISCAN-Colon	1	Knudsen, A. B. (2016) [31]	US	45-75	Lifetime	100%	28.0/1000	9.0/1000
			US	50-75	Lifetime	100%	28.0/1000	10.0/1000
			US	55-75	Lifetime	100%	28.0/1000	11.0/1000
			US	45-80	Lifetime	100%	28.0/1000	8.0/1000
			US	50-80	Lifetime	100%	28.0/1000	9.0/1000
			US	55-80	Lifetime	100%	28.0/1000	10.0/1000
	2	Meester, R. G. S. (2018) [36]	US White Female	45-75	Lifetime	100%	21.9/1000	7.9/1000
			US Black Female	45-75	Lifetime	100%	28.4/1000	9.4/1000
			US White Male	45-75	Lifetime	100%	27.2/1000	9.2/1000
			US Black Male	45-75	Lifetime	100%	29.6/1000	9.6/1000
			US White Female	50-75	Lifetime	100%	21.9/1000	8.9/1000
			US Black Female	50-75	Lifetime	100%	28.4/1000	10.4/1000
			US White Male	50-75	Lifetime	100%	27.2/1000	10.2/1000
			US Black Male	50-75	Lifetime	100%	29.6/1000	10.6/1000
			US White Female	55-75	Lifetime	100%	21.9/1000	8.9/1000
			US Black Female	55-75	Lifetime	100%	28.4/1000	11.4/1000
			US White Male	55-75	Lifetime	100%	27.2/1000	11.2/1000

			US Black Male	55-75	Lifetime	100%	29.6/1000	12.6/1000
			US White Female	45-80	Lifetime	100%	21.9/1000	6.9/1000
			US Black Female	45-80	Lifetime	100%	28.4/1000	8.4/1000
			US White Male	45-80	Lifetime	100%	27.2/1000	8.2/1000
			US Black Male	45-80	Lifetime	100%	29.6/1000	8.6/1000
			US White Female	50-80	Lifetime	100%	21.9/1000	6.9/1000
			US Black Female	50-80	Lifetime	100%	28.4/1000	9.4/1000
			US White Male	50-80	Lifetime	100%	27.2/1000	8.2/1000
			US Black Male	50-80	Lifetime	100%	29.6/1000	9.6/1000
			US White Female	55-80	Lifetime	100%	21.9/1000	7.9/1000
			US Black Female	55-80	Lifetime	100%	28.4/1000	10.4/1000
			US White Male	55-80	Lifetime	100%	27.2/1000	10.2/1000
			US Black Male	55-80	Lifetime	100%	29.6/1000	11.6/1000
	3	Gini, A. (2021) [25]	Finnish	60-69	4.5 years	69%	24.0/100000	22.0/100000
CRC-AIM	4	Piscitello, A. (2020) [40]	US	45-75	Lifetime	100%	31.7/1000	8.7/1000
			US	45-80	Lifetime	100%	31.7/1000	7.6/1000
			US	50-75	Lifetime	100%	31.7/1000	10.2/1000
			US	50-80	Lifetime	100%	31.7/1000	8.5/1000
			US	55-75	Lifetime	100%	31.7/1000	11.5/1000
			US	55-80	Lifetime	100%	31.7/1000	10.3/1000

Table S6. Characteristics of simulation models on single FS screening from age of 50–75 years.

Model	Study No.	Study	Simulate population	Screening age	Simulation period/Follow-up time (years)	Adherence rate in screening group (%)	CRC mortality of no screening group	CRC mortality of screening group
SimCRC	1	DeYoreo, M. (2020) [24]	UK	55-64	17 years	71.1%	73.0/100000	44.0/100000
	2	Rutter, C. M. (2016) [42]	UK	55-64	10 years	71.1%	52.0/100000	33.0/100000
CRC-SPIN	1	DeYoreo, M. (2020) [24]	UK	55-64	17 years	71.1%	70.0/100000	33.0/100000

	2	Rutter, C. M. (2016) [42]	UK	55-64	10 years	71.1%	38.0/100000	21.0/100000
MISCAN-Colon	1	DeYoreo, M. (2020) [24]	UK	55-64	17 years	71.1%	54.0/100000	26.0/100000
	2	Rutter, C. M. (2016) [42]	UK	55-64	10 years	71.1%	37.0/100000	25.0/100000
	3	Buskermolen, M. (2019) [20]	Norwegian	50-79	15 years	100%	9.0/1000	4.0/1000
	4	Buskermolen, M. (2018) [21]	Norwegian	50-65	18 years	63%	56.5/100000	41.0/100000
	5	Gini, A. (2021) [25]	Italian	55-64	11.4 years	58.30%	65.0/100000	46.0/100000
			Norwegian	50-64	10.9 years	63%	41.0/100000	29.0/100000
	6	Heijnsdijk, E. A. M. (2019) [29]	Dutch	55-75	15 years	73%	12.0/1000	9.0/1000

Table S7. Characteristics of simulation models on 10 yearly colonoscopy screening from age of 55–75 years.

**Model**	**Study No.**	**Study**	**Simulate population**	**Screening age**	**Simulation period/Follow-up time (years)**	**Adherence rate in screening group (%)**	**CRC mortality of no screening group**	**CRC mortality of screening group**
ASCCA	1	Vleugels, J. L. A. (2017) [43]	Dutch	55-75	Lifetime	22%	28.2/1000	20.5/1000
SimCRC	2	Knudsen, A. B. (2016) [31]	US	55-75	Lifetime	100%	28.0/1000	5.0/1000
	3	Knudsen, A. B. (2021) [30]	US	55-75	Lifetime	100%	34.0/1000	6.0/1000
	4	Meester, R. G. S. (2018) [36]	US White Female	55-75	Lifetime	100%	25.7/1000	3.7/1000
			US Black Female	55-75	Lifetime	100%	30.0/1000	6.0/1000
			US White Male	55-75	Lifetime	100%	31.0/1000	5.0/1000
			US Black Male	55-75	Lifetime	100%	27.0/1000	5.0/1000
CRC-SPIN	2	Knudsen, A. B. (2016) [31]	US	55-75	Lifetime	100%	27.0/1000	3.0/1000
	3	Knudsen, A. B. (2021) [30]	US	55-75	Lifetime	100%	32.0/1000	6.0/1000
MISCAN-Colon	2	Knudsen, A. B. (2016) [31]	US	55-75	Lifetime	100%	28.0/1000	7.0/1000
	3	Knudsen, A. B. (2021) [30]	US	55-75	Lifetime	100%	34.0/1000	10.0/1000
	4		US White Female	55-75	Lifetime	100%	21.9/1000	4.9/1000

		Meester, R. G. S. (2018) [36]	US Black Female	55-75	Lifetime	100%	28.4/1000	7.4/1000
			US White Male	55-75	Lifetime	100%	27.2/1000	6.2/1000
			US Black Male	55-75	Lifetime	100%	29.6/1000	7.6/1000
	5	Cenin, D. R. (2020) [22]	Australian	54-74	Lifetime 40-100	100%	29.0/1000	10.0/1000
			Australian	54-74	Lifetime 40-100	Realistic adherence	29.0/1000	21.0/1000
	6	Gini, A. (2021) [62]	Dutch	55-75	2018–50	71.3%	26.5/1000	16.8/1000
CRC-AIM	7	Piscitello, A. (2020) [40]	US	55-75	Lifetime	100%	31.7/1000	4.9/1000

Table S8. Characteristics of simulation models on 5 yearly flexible sigmoidoscopy (FS) screening from age of 55–75 years.

**Model**	**Study No.**	**Study**	**Simulate population**	**Screening age**	**Simulation period/Follow-up time (years)**	**Adherence rate in screening group (%)**	**CRC mortality of no screening group**	**CRC mortality of screening group**
SimCRC	1	Knudsen, A. B. (2016) [31]	US	55-75	Lifetime	100%	28.0/1000	9.0/1000
	2	Knudsen, A. B. (2021) [30]	US	55-75	Lifetime	100%	34.0/1000	11.0/1000
	3	Meester, R. G. S. (2018) [36]	US White Female	55-75	Lifetime	100%	25.7/1000	8.7/1000
			US Black Female	55-75	Lifetime	100%	30.0/1000	11.0/1000
			US White Male	55-75	Lifetime	100%	31.0/1000	9.0/1000
			US Black Male	55-75	Lifetime	100%	27.0/1000	9.0/1000
CRC-SPIN	1	Knudsen, A. B. (2016) [31]	US	55-75	Lifetime	100%	27.0/1000	11.0/1000
	2	Knudsen, A. B. (2021) [30]	US	55-75	Lifetime	100%	32.0/1000	11.0/1000
MISCAN-Colon	1	Knudsen, A. B. (2016) [31]	US	55-75	Lifetime	100%	28.0/1000	9.0/1000
	2	Knudsen, A. B. (2021) [30]	US	55-75	Lifetime	100%	34.0/1000	12.0/1000
	3	Meester, R. G. S. (2018) [36]	US White Female	55-75	Lifetime	100%	21.9/1000	7.9/1000
			US Black Female	55-75	Lifetime	100%	28.4/1000	10.4/1000
			US White Male	55-75	Lifetime	100%	27.2/1000	8.2/1000
			US Black Male	55-75	Lifetime	100%	29.6/1000	10.6/1000
CRC-AIM	4	Piscitello, A. (2020) [40]	US	55-75	Lifetime	100%	31.7/1000	14.2/1000

Table S9. Egger's Test for publication bias of simulation modelling studies on CRC-specific mortality reduction.

Outcomes	No. of studies	No. of scenarios	Bias	Intercept	*t*	*P value*
Biennial FIT, 55-75	15	27	-2.8968	0.1458	-3.19	**0.004**
Biennial FOBT, 45-80	4	73	-6.0698	1.1955	-14.07	**<0.001**
Single FS, 50-75	6	11	-0.3413	-0.4072	-0.40	0.698
10 yearly CS, 55-75	7	19	-6.6412	1.4527	-13.50	**<0.001**
5 yearly FS, 55-75	4	15	-2.6840	-0.0790	-2.76	**0.016**

Table S10. Meta regression of simulation models on CRC-specific mortality reduction by 10 yearly colonoscopy screening from age of 55–75 years.

Factor	I^2(%)	Estimate	SE	$P_{regression}$
Adherence	19.36	1.1823	0.2396	**<0.001**

Table S11. Subgroup analysis of simulation models on CRC-specific mortality reduction by 10 yearly colonoscopy screening from age of 55–75 years.

Subgroups	Pooled estimate	No. of scenarios	I^2(%)	P_{sub}
Adherence				
Perfect adherence (100%)	0.21 (0.17, 0.27)	16	0	**<0.001**
Realistic adherence	0.70 (0.50, 0.97)	3	0	

Table S12. Subgroup analysis of simulation models on CRC-specific mortality reduction by biennial gFOBT screening from age of 45–80 years.

Subgroups	Pooled estimate	No. of scenarios	I^2(%)	P_{sub}
Adherence				
Perfect adherence (100%)	0.30 (0.28, 0.33)	72	0	**<0.001**
Realistic adherence (69%)	0.92 (0.51, 1.63)	1	—	

Table S13. Subgroup analysis of simulation models on CRC-specific mortality reduction by single FS screening from age of 50–75 years.

Subgroups	Pooled estimate	No. of scenarios	I^2(%)	P_{sub}
Simulate population				
UK	0.56 (0.48, 0.66)	6	0	**0.004**
Italian, Norwegian, and Dutch	0.70 (0.61, 0.80)	5	0	
Model				
MISCAN-Colon	0.66 (0.56, 0.77)	7	0	0.105
SimCRC, and CRC-SPIN	0.56 (0.46, 0.70)	4	0	

Table S14. Characteristics of simulation models on all-cause mortality reduction of CRC screening.

Model	Article ID	Study	Simulate population	Screening age	Follow-up time	Screening methods	Adherence rate in screening group (%)	CRC risk	All-cause mortality reduction
MISCAN-Colon	5	Buskermolen, M. (2019) [20]	Norwegian	50-79	15 years	Biennial FIT	100	3%*	1.10%
			Norwegian	50-79	15 years	Annual FIT	100	3%*	1.30%
			Norwegian	50-79	15 years	Single FS	100	3%*	1.10%
			Norwegian	50-79	15 years	Single colonoscopy	100	3%*	1.40%
	23	Heijnsdijk, E. A. M. (2019) [29]	Dutch	55-75	15 years	Single FS	73	Average	0.62%

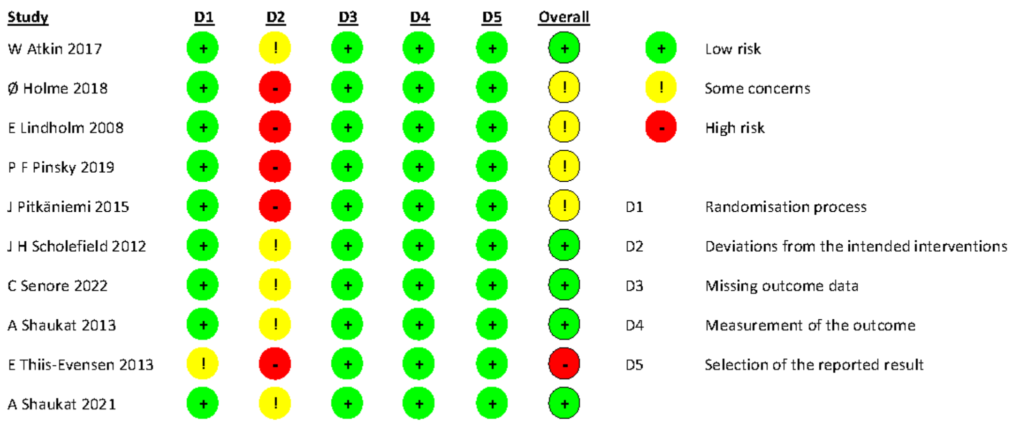

Figure S1. Quality assessment of included RCTs articles [48-57].

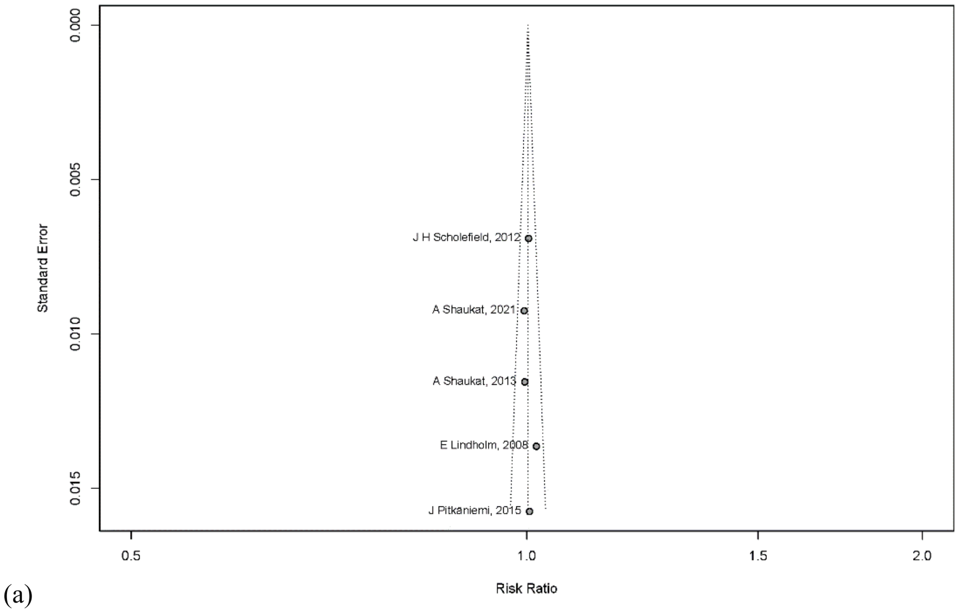

(a)

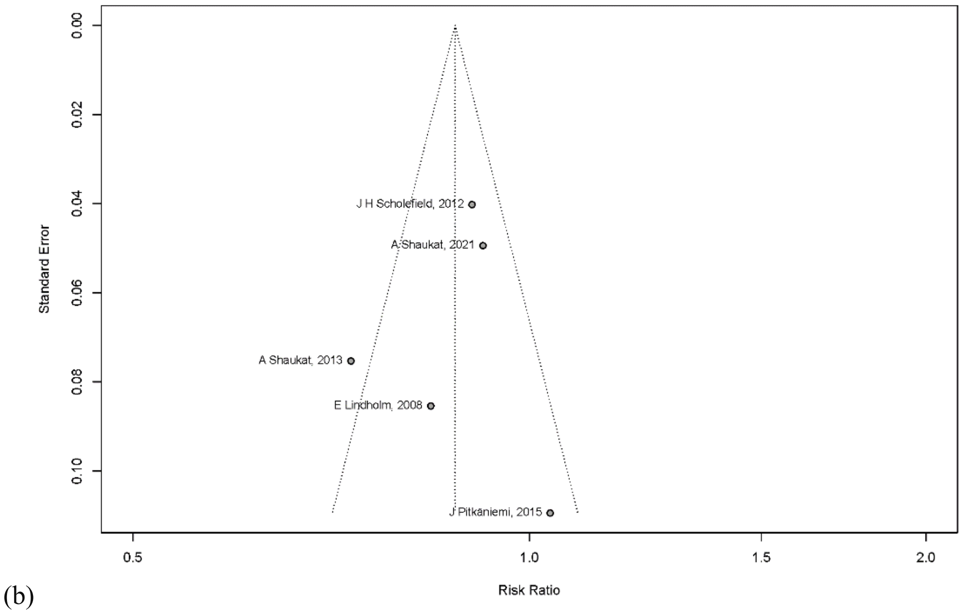

(b)

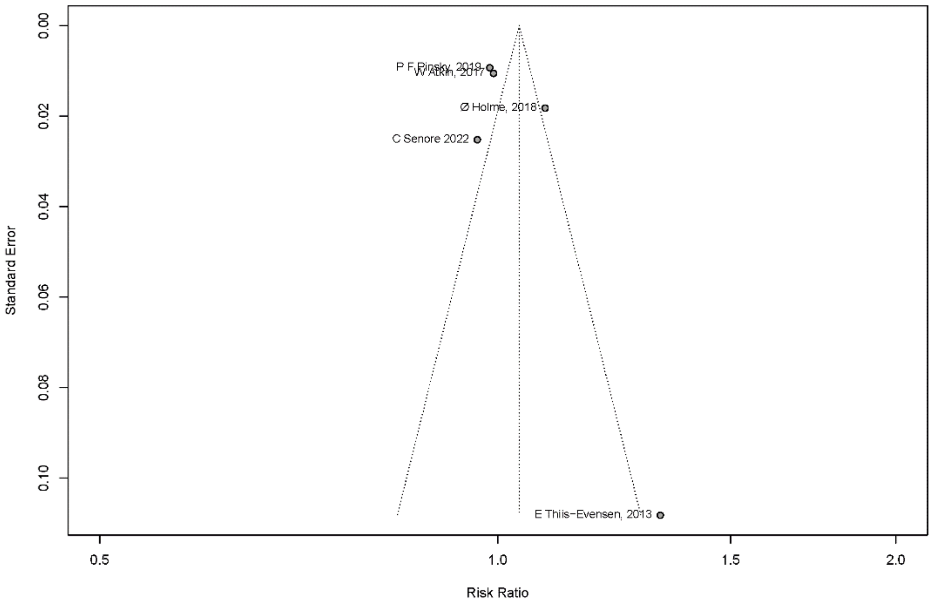

(c)

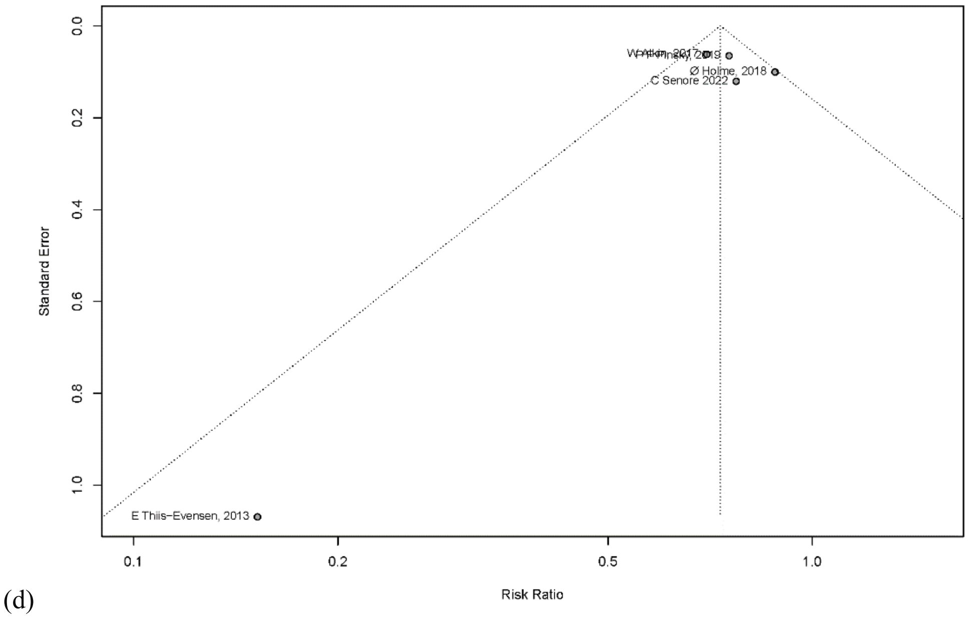

(d)

Figure S2. Funnel plots for assessing publication bias (Intention-to-treat analyses). a. All-cause mortality rate ratio on FOBT screening programs [49,51,52,54,56]; b. CRC-specific mortality rate ratio on FOBT screening programs [49,51,52,54,56]; c. All-cause mortality rate ratio on FS screening programs [48,50,53,55,57]; d. CRC-specific mortality rate ratio on FS screening programs [48,50,53,55,57].

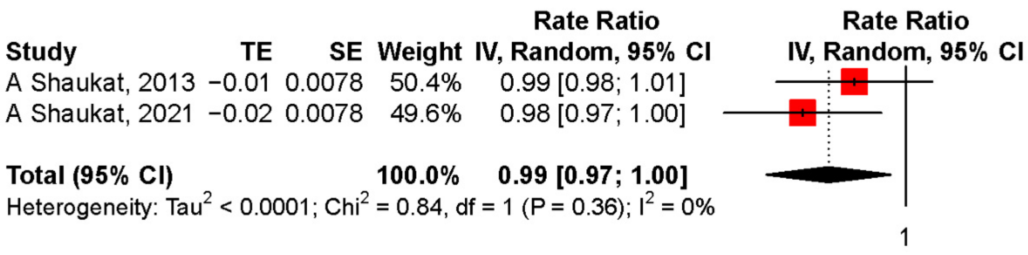

(a)

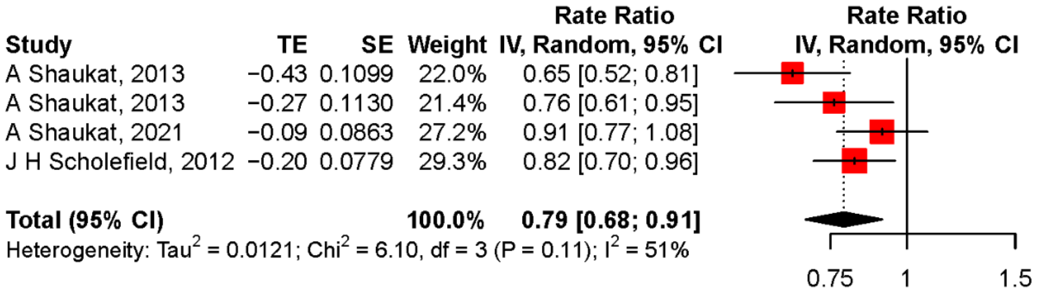

(b)

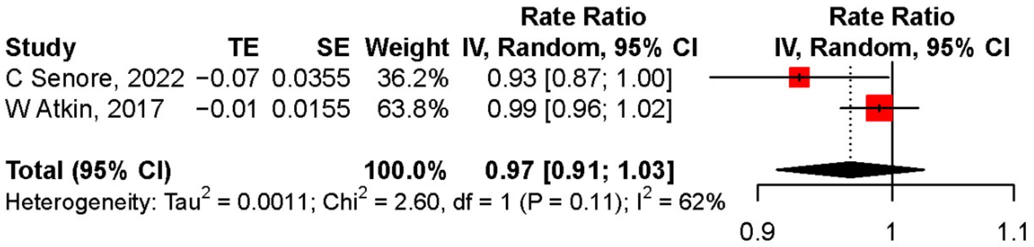

(c)

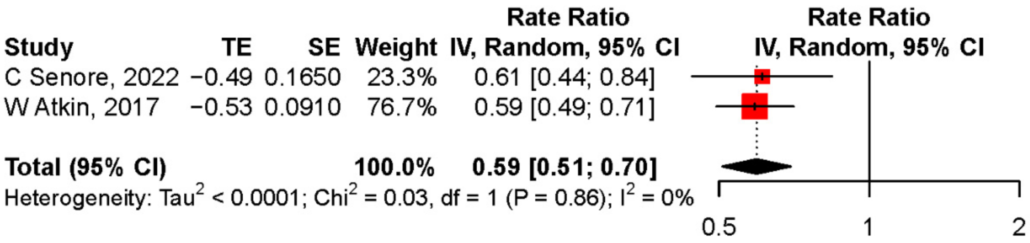

(d)

Figure S3. Forest plots of the compliance-adjusted analysis. a. All-cause mortality rate ratio on FOBT screening programs [51,52]; b. CRC-specific mortality rate ratio on FOBT screening programs [49,51,52]; c. All-cause mortality rate ratio on FS screening programs [48,50]; d. CRC-specific mortality rate ratio on FS screening programs [48,50].

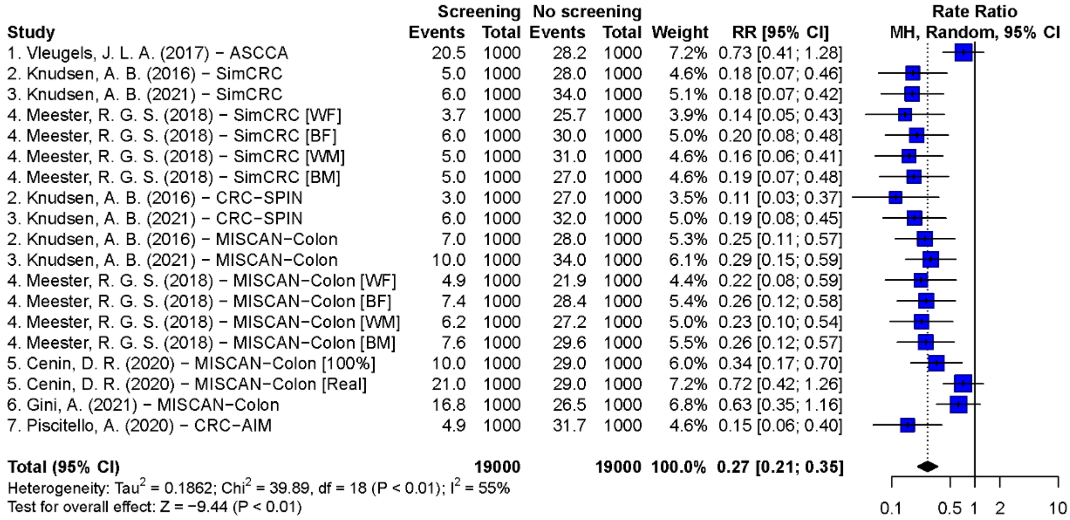

Figure S4. Forest plots of the CRC-specific mortality rate ratio on 10 yearly colonoscopy screening from age of 55–75 years [22,30,31,36,40,43,62].

Study	Screening Events	Screening Total	No screening Events	No screening Total	Weight	RR [95% CI]
1. Knudsen, A. B. (2016) − SimCRC	9.0	1000	28.0	1000	6.1%	0.32 [0.15; 0.68]
2. Knudsen, A. B. (2021) − SimCRC	11.0	1000	34.0	1000	7.4%	0.32 [0.16; 0.63]
3. Meester, R. G. S. (2018) − SimCRC [WF]	8.7	1000	25.7	1000	5.8%	0.34 [0.16; 0.73]
3. Meester, R. G. S. (2018) − SimCRC [BF]	11.0	1000	30.0	1000	7.2%	0.37 [0.18; 0.73]
3. Meester, R. G. S. (2018) − SimCRC [WM]	9.0	1000	31.0	1000	6.2%	0.29 [0.14; 0.61]
3. Meester, R. G. S. (2018) − SimCRC [BM]	9.0	1000	27.0	1000	6.0%	0.33 [0.16; 0.71]
1. Knudsen, A. B. (2016) − CRC−SPIN	11.0	1000	27.0	1000	7.0%	0.41 [0.20; 0.82]
2. Knudsen, A. B. (2021) − CRC−SPIN	11.0	1000	32.0	1000	7.3%	0.34 [0.17; 0.68]
1. Knudsen, A. B. (2016) − MISCAN−Colon	9.0	1000	28.0	1000	6.1%	0.32 [0.15; 0.68]
2. Knudsen, A. B. (2021) − MISCAN−Colon	12.0	1000	34.0	1000	7.9%	0.35 [0.18; 0.68]
3. Meester, R. G. S. (2018) − MISCAN−Colon [WF]	7.9	1000	21.9	1000	5.2%	0.36 [0.16; 0.81]
3. Meester, R. G. S. (2018) − MISCAN−Colon [BF]	10.4	1000	28.4	1000	6.8%	0.37 [0.18; 0.74]
3. Meester, R. G. S. (2018) − MISCAN−Colon [WM]	8.2	1000	27.2	1000	5.6%	0.30 [0.14; 0.65]
3. Meester, R. G. S. (2018) − MISCAN−Colon [BM]	10.6	1000	29.6	1000	6.9%	0.36 [0.18; 0.72]
4. Piscitello, A. (2020) − CRC−AIM	14.2	1000	31.7	1000	8.7%	0.45 [0.24; 0.83]
**Total (95% CI)**		**15000**		**15000**	**100.0%**	**0.35 [0.29; 0.42]**

Heterogeneity: $Tau^2 = 0.0010$; $Chi^2 = 1.40$, df = 14 (P = 1.00); $I^2 = 0\%$
Test for overall effect: Z = −11.17 (P < 0.01)

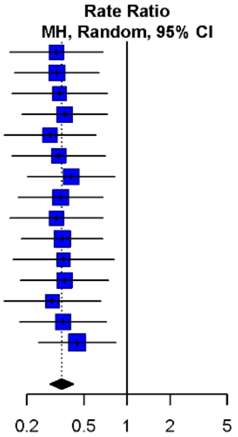

Figure S5. Forest plots of the CRC-specific mortality rate ratio on 5 yearly FS screening from age of 55–75 years [30,31,36,40].

Study	Screening Events	Screening Total	No screening Events	No screening Total	Weight	RR [95% CI]
1. Deibel, A. (2021) − CMOST [100%]	11.6	1000	25.6	1000	2.5%	0.45 [0.23; 0.90]
1. Deibel, A. (2021) − CMOST [<100%]	15.5	1000	25.6	1000	2.9%	0.61 [0.32; 1.13]
2. Piscitello, A. (2020) − CRC−AIM	10.7	1000	31.7	1000	2.5%	0.34 [0.17; 0.67]
3. Knudsen, A. B. (2016) − SimCRC	8.0	1000	28.0	1000	2.2%	0.29 [0.13; 0.62]
4. Knudsen, A. B. (2021) − SimCRC	10.0	1000	34.0	1000	2.5%	0.29 [0.15; 0.59]
5. Meester, R. G. S. (2018) − SimCRC [WF]	7.7	1000	25.7	1000	2.1%	0.30 [0.13; 0.67]
5. Meester, R. G. S. (2018) − SimCRC [BF]	10.0	1000	30.0	1000	2.4%	0.33 [0.16; 0.68]
5. Meester, R. G. S. (2018) − SimCRC [WM]	8.0	1000	31.0	1000	2.2%	0.26 [0.12; 0.56]
5. Meester, R. G. S. (2018) − SimCRC [BM]	8.0	1000	27.0	1000	2.1%	0.30 [0.14; 0.65]
3. Knudsen, A. B. (2016) − CRC−SPIN	8.0	1000	27.0	1000	2.1%	0.30 [0.14; 0.65]
4. Knudsen, A. B. (2021) − CRC−SPIN	10.0	1000	32.0	1000	2.5%	0.31 [0.15; 0.63]
3. Knudsen, A. B. (2016) − MISCAN−Colon	11.0	1000	28.0	1000	2.5%	0.39 [0.20; 0.78]
4. Knudsen, A. B. (2021) − MISCAN−Colon	14.0	1000	34.0	1000	2.9%	0.41 [0.22; 0.76]
5. Meester, R. G. S. (2018) − MISCAN−Colon [WF]	8.9	1000	21.9	1000	2.2%	0.41 [0.19; 0.88]
5. Meester, R. G. S. (2018) − MISCAN−Colon [BF]	10.4	1000	28.4	1000	2.5%	0.37 [0.18; 0.74]
5. Meester, R. G. S. (2018) − MISCAN−Colon [WM]	10.2	1000	27.2	1000	2.4%	0.38 [0.18; 0.77]
5. Meester, R. G. S. (2018) − MISCAN−Colon [BM]	10.6	1000	29.6	1000	2.5%	0.36 [0.18; 0.72]
6. Cenin, D. R. (2020) − MISCAN−Colon [100%]	15.0	1000	29.0	1000	2.9%	0.52 [0.28; 0.96]
6. Cenin, D. R. (2020) − MISCAN−Colon [Real]	22.0	1000	29.0	1000	3.3%	0.76 [0.44; 1.31]
7. Peterse, E. F. P. (2018) − MISCAN−Colon	17.0	1000	45.0	1000	3.3%	0.38 [0.22; 0.66]
8. van der Meulen, M. P. (2017) − MISCAN−Colon [NLM]	9.6	1000	20.7	1000	2.2%	0.46 [0.22; 0.99]
8. van der Meulen, M. P. (2017) − MISCAN−Colon [NLF]	10.6	1000	18.5	1000	2.3%	0.57 [0.27; 1.21]
9. Cenin, D. (2022) − MISCAN−Colon	4.0	1000	11.0	1000	1.2%	0.36 [0.12; 1.14]
10. Babela, R. (2021) − MISCAN−Colon	10.7	1000	13.0	1000	2.1%	0.82 [0.37; 1.84]
11. Heinävaara, S. (2022) − MISCAN−Colon	13.0	1000	23.0	1000	2.6%	0.57 [0.29; 1.11]
12. Lew, J. B. (2018) − Policy 1−Bowel [100%]	6.2	1000	28.8	1000	1.9%	0.22 [0.09; 0.51]
12. Lew, J. B. (2018) − Policy 1−Bowel [60%]	13.0	1000	28.8	1000	2.7%	0.45 [0.24; 0.86]
12. Lew, J. B. (2018) − Policy 1−Bowel [40%]	17.6	1000	28.8	1000	3.1%	0.61 [0.34; 1.10]
13. Lew, J. B. (2018) − Policy 1−Bowel [100%]	6.1	100000	23.0	100000	1.8%	0.27 [0.11; 0.65]
13. Lew, J. B. (2018) − Policy 1−Bowel [57%]	11.3	100000	23.0	100000	2.4%	0.49 [0.24; 1.00]
13. Lew, J. B. (2018) − Policy 1−Bowel [29%]	14.7	100000	23.0	100000	2.7%	0.64 [0.33; 1.23]
14. Lew, J. B. (2017) − Policy 1−Bowel [40%]	14.6	100000	22.9	100000	2.7%	0.64 [0.33; 1.23]
14. Lew, J. B. (2017) − Policy 1−Bowel [60%]	11.0	100000	22.9	100000	2.4%	0.48 [0.23; 0.99]
14. Lew, J. B. (2017) − Policy 1−Bowel [70%]	9.5	100000	22.9	100000	2.2%	0.41 [0.19; 0.88]
15. Lew, J. B. (2022) − Policy 1−Bowel [23%]	146.0	10000	191.0	10000	5.9%	0.76 [0.62; 0.95]
15. Lew, J. B. (2022) − Policy 1−Bowel [42%]	115.0	10000	191.0	10000	5.7%	0.60 [0.48; 0.76]
16. Heisser, T. (2022) − Multistate Markov [Men]	11.0	1000	61.0	1000	2.8%	0.18 [0.10; 0.34]
16. Heisser, T. (2022) − Multistate Markov [Women]	12.0	1000	48.0	1000	2.8%	0.25 [0.13; 0.47]
**Total (95% CI)**		**650000**		**650000**	**100.0%**	**0.43 [0.37; 0.49]**

Heterogeneity: $Tau^2 = 0.0731$; $Chi^2 = 64.91$, df = 37 (P < 0.01); $I^2 = 43\%$
Test for overall effect: Z = −11.98 (P < 0.01)

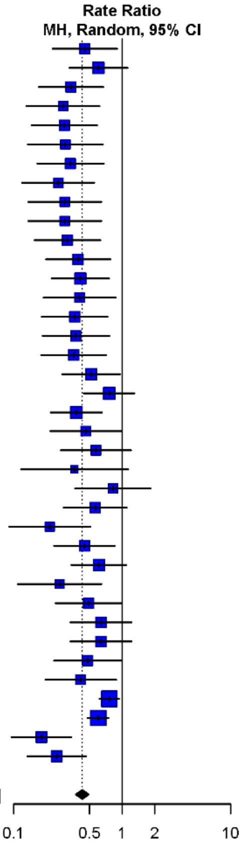

(a)

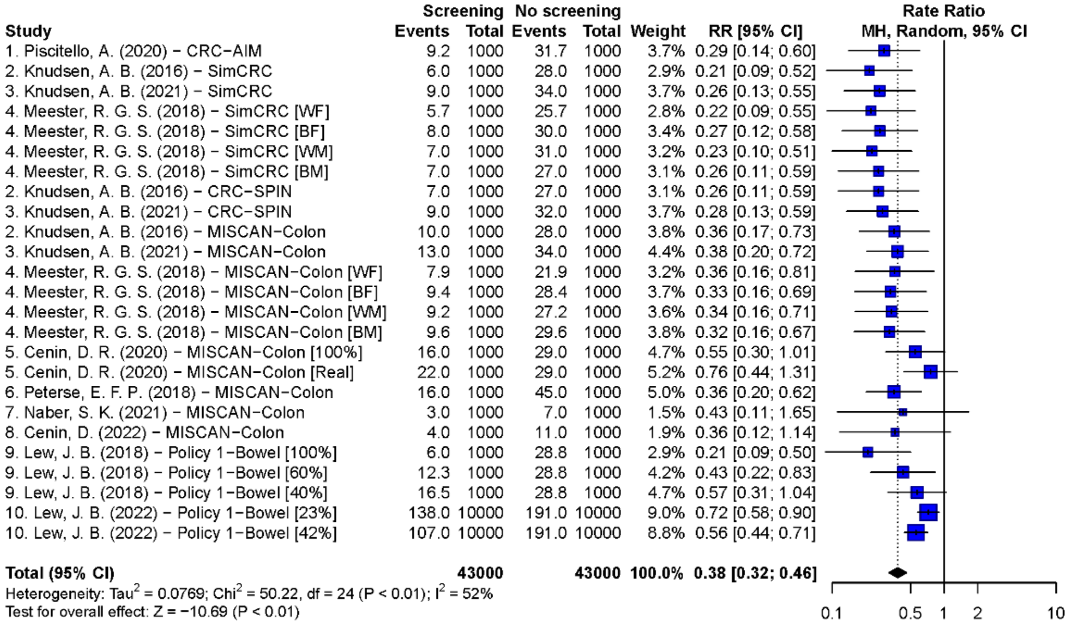

(b)

Figure S6. Forest plots of the CRC-specific mortality rate ratio on biennial FIT screening. a. from age of 50–75 years [13,22,23,30,31,33,34,36,37,39,40,58,60,63,74,75]; b. from age of 45–75 years [13,22,30,31,36,39,40,60,64,75].

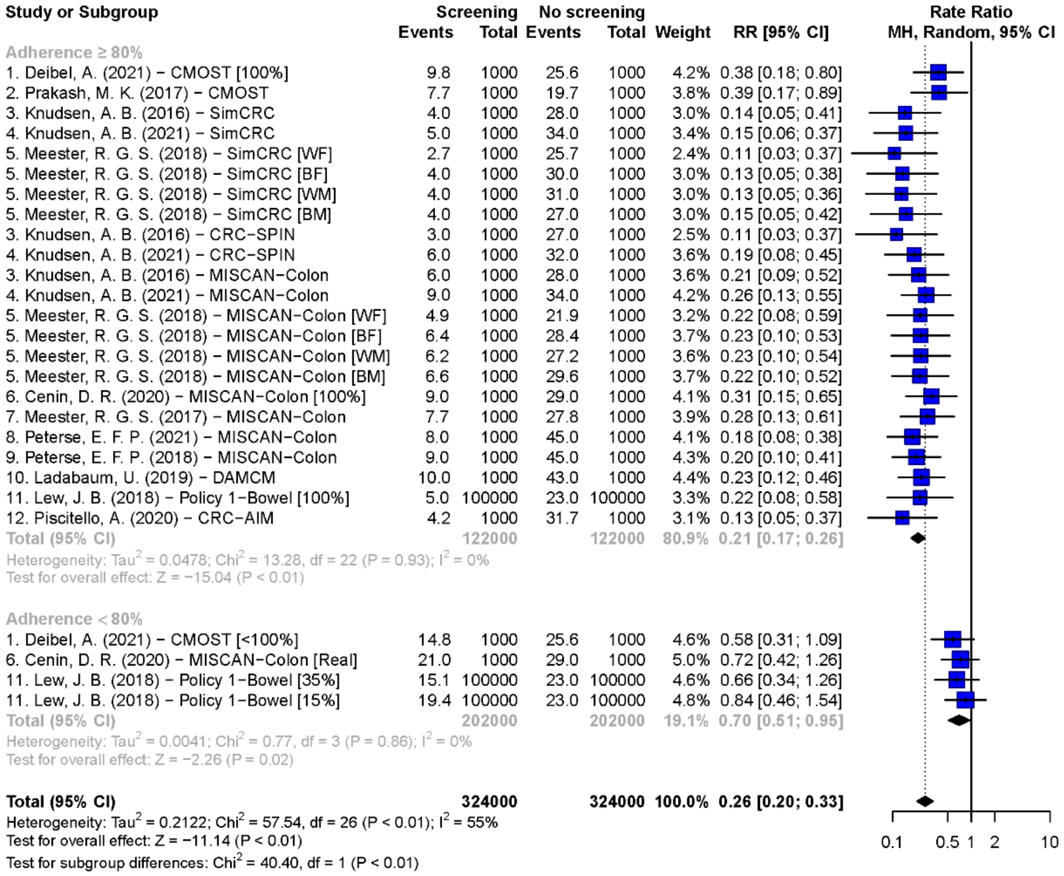

(a)

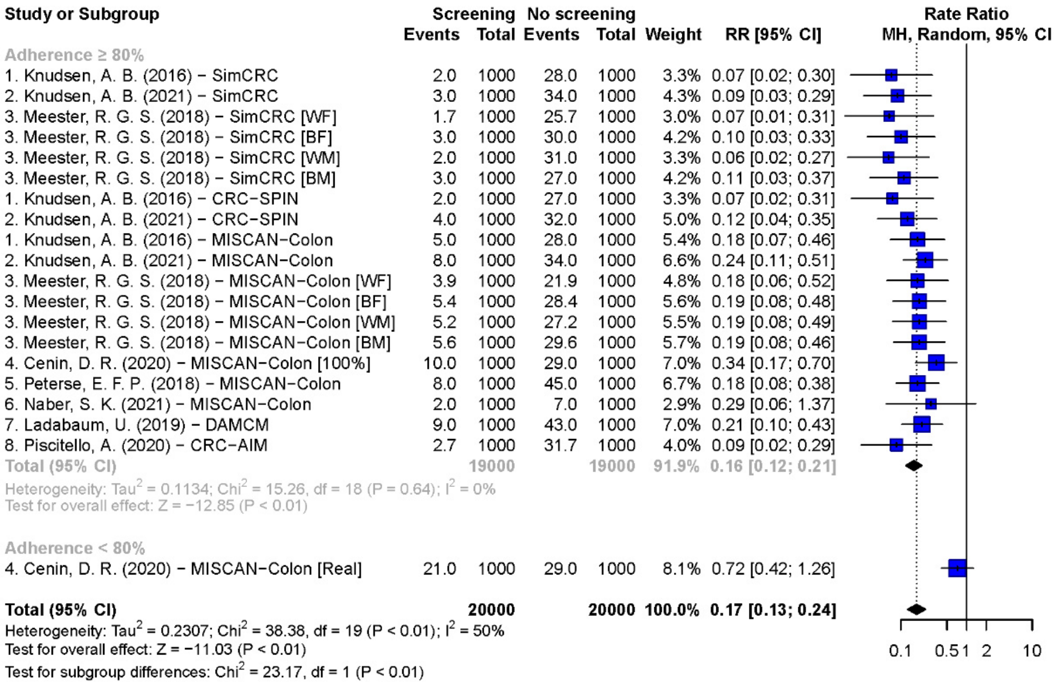

(b)

Figure S7. Forest plots of the CRC-specific mortality rate ratio on 10 yearly colonoscopy screening. a. from age of 50–75 years [13,22,23,30-32,35,36,38-41]; b. from age of 45–75 years [22,30-32,36,39,40,64].

*DAMCM: Decision analytic Markov cohort model.

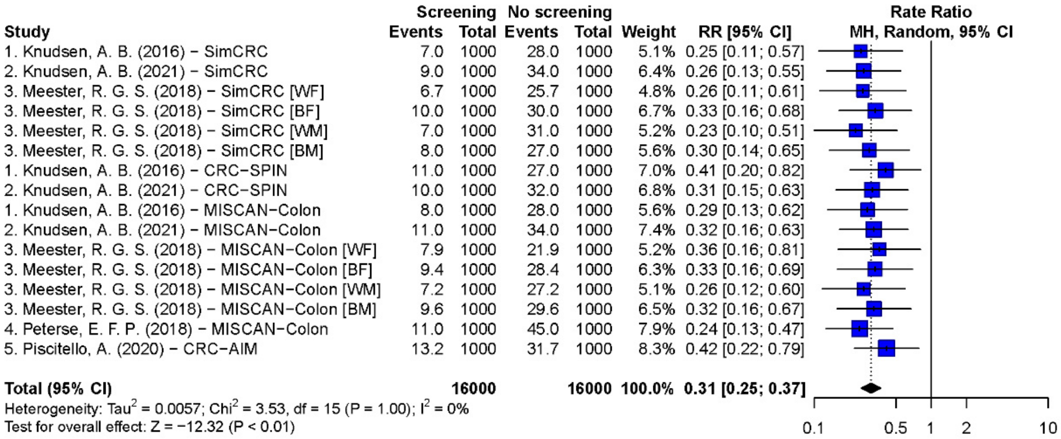

(a)

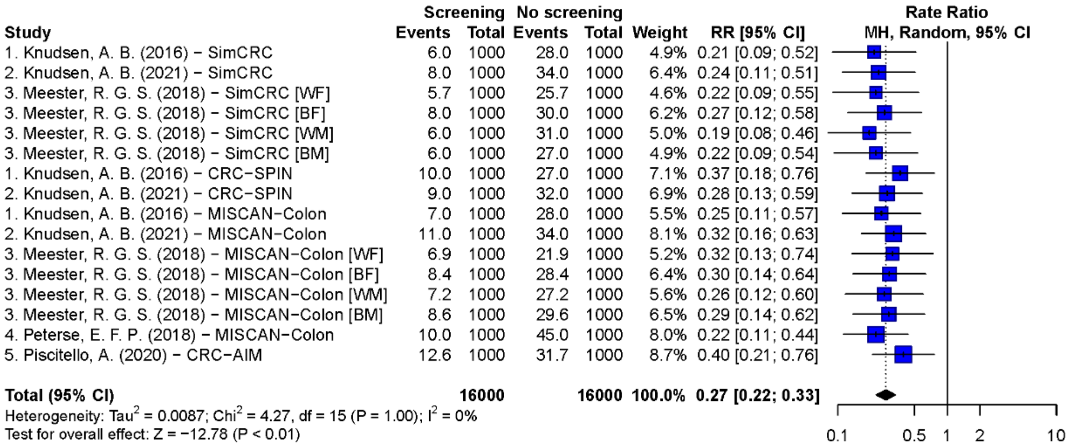

(b)

Figure S8. Forest plots of the CRC-specific mortality rate ratio on 5 yearly FS screening. a. from age of 50–75 years [30,31,36,39,40]; b. from age of 45–75 years [30,31,36,39,40].
